# Gene expression biomarkers differentiate overall survival of colorectal cancer upon targeted therapies

**DOI:** 10.21203/rs.3.rs-4047331/v1

**Published:** 2024-03-11

**Authors:** Akram Yazdani, Azam Yazdani, Raul Mendez-Giraldez, Gianluigi Pillonetto, Esmat Samiei, Reza Hadi, Heinz-Josef Lenz, Alan Venook, Ahmad Samiei, Andrew Nixon, Joseph Lucci, Scott Kopetz, Monica Bertagnolli, Charles Perou, Federico Innocenti

**Affiliations:** University of Texas, Houston medical center; Brigham & Women’s Hospital; National Institute of Environmental Health Sciences, Durham; University of Padova; Tehran; University of Science and Technology of Iran; USC; University of California San Fran*cis*co; Brigham & Women’s Hospital; Duke University; The University of Texas Health SCience Center; The University of Texas MD Anderson Cancer Center; Brigham and Women’s Hospital; Lineberger Comprehensive Cancer Center; AbbVie

## Abstract

While monoclonal antibody-based targeted therapies have substantially improved progression-free survival in cancer patients, the variability in individual responses poses a significant challenge in patient care. Therefore, identifying cancer subtypes and their associated biomarkers is required for assigning effective treatment. In this study, we integrated genotype and pre-treatment tissue RNA-seq data and identified biomarkers causally associated with the overall survival (OS) of colorectal cancer (CRC) patients treated with either cetuximab or bevacizumab. We performed enrichment analysis for specific consensus molecular subtypes (CMS) of colorectal cancer and evaluated differential expression of identified genes using paired tumor and normal tissue from an external cohort. In addition, we replicated the causal effect of these genes on OS using validation cohort and assessed their association with the Cancer Genome Atlas Program data as an external cohort. One of the replicated findings was *WDR62*, whose overexpression shortened OS of patients treated with cetuximab. Enrichment of its over expression in CMS1 and low expression in CMS4 suggests that patients with CMS4 subtype may drive greater benefit from cetuximab. In summary, this study highlights the importance of integrating different omics data for identifying promising biomarkers specific to a treatment or a cancer subtype.

## Introduction

While targeted therapies utilizing monoclonal antibodies have improved progression-free survival in patients, the pursuit of more effective cancer treatments remains a top priority. What complicates this pursuit is the diverse range of patient responses to existing therapies, which manifests as a significant obstacle in understanding the underlying variability in clinical outcomes^1^. Therefore, the key to addressing this challenge is identifying cancer subtypes and their associated biomarkers, allowing for pre*cis*ion in treatment strategies and ultimately leading to enhanced patient outcomes and a personalized approach to cancer care.

In the context of colorectal cancer (CRC), the transcriptomics-based consensus molecular subtype (CMS) classification has been an instrument to reveal molecular signatures closely associated with prognosis and treatment response in CRC^2^. This classification categorizes CRC into four CMS groups, CMS1 (microsatellite instability immune), CMS2 (canonical), CMS3 (metabolic), and CMS4 (mesenchymal), based on the underlying biology of the disease rather than relying solely on outcomes^3^. Nevertheless, several key aspects of CRC progression and response to treatment remain unresolved^4^. Therefore, linking these subtypes to clinical outcomes and identifying treatment-specific biomarkers for each subtype can hold promise in contributing to the development of targeted therapies. Ultimately, this can advance pre*cis*ion medicine in the management of CRC.

Here, we analyzed data from a randomized phase III trial (CALGB/SWOG 80405) involving 1,284 patients with KRAS wild-type advanced or metastatic CRC. These patients were treated with either cetuximab or bevacizumab in combination with chemotherapy (FOLFOX or FOLFIRI). Cetuximab targets the epidermal growth factor receptor (EGFR) and influences immune cells, including natural killer (NK) cells and macrophages^5^. In contrast, bevacizumab targets vascular endothelial growth factor (VEGF) and inhibits tumor angiogenesis. The clinical outcomes of this study did not reveal a significant difference in overall survival (OS) between these two treatments when used as first-line therapy^6^. However, given the fact that these treatments impact distinct biological pathways, different mechanisms play roles in the response or resistance to these therapies. Hence, it is imperative to delve into these mechanisms at the molecular level and associate them with specific cancer subtypes.

To investigate the influence of gene expressions on treatment response, we segmented our dataset into a discovery cohort and a validation cohort, based on the availability of gene expression data. In the discovery cohort, comprising samples with RNA-seq data, we conducted one-sample Mendelian randomization (MR) analysis, integrating germline genotype, RNA-seq, and treatment-specific overall survival (OS) data. In the validation cohort, which included patients with available germline genotype data and clinical outcomes, including OS, we performed a two-sample MR analysis to replicate the results obtained in the discovery cohort. Furthermore, we sought to replicate our findings using external cohorts, including The Cancer Genome Atlas (TCGA) data and the GSE146889 dataset sourced from the Gene Expression Omnibus database. To elucidate a more pre*cis*e understanding of how these biomarkers influence treatment responses within distinct patient groups, we linked the identified biomarkers with CRC CMS (consensus molecular subtypes).

## Results

In our investigation of treatment-specific biomarkers for CRC, we focused on cetuximab and bevacizumab monoclonal antibodies used in the randomized phase III trial CALGB/SWOG 80405. The trial was designed to compare cetuximab, bevacizumab, or cetuximab + bevacizumab, each plus chemotherapy as first-line therapy in KRAS wild-type advanced or metastatic CRC. The discovery cohort encompassed 273 patients with pre-treatment tumor primary tissue RNA-seq and germline genotype data (**Figure S1–2**). The validation cohort included 602 patients with germline genotype data (**Figure S1–2**). In both cohorts, we had records of OS and conducted an MR technique, one- and two-sample MR to identify genes with causal effect on treatment-specific OS. In one-sample MR analysis, we assessed the causal effect of the genes associated with OS. We replicated the identified associations using TCGA data. To reproduce the causal impact of genes on OS, we predicted gene expression levels by germline genotype data in the validation cohort and eQTL summary statistics from the discovery cohort^7^ and performed a two-sample MR analysis. In addition, we investigated the differential expression of findings in colorectal tumors vs. normal tissue using the GSE146889 data from the Gene Expression Omnibus database. We finally linked the findings to CMS to investigate the genetic basis underpinning CMS^3^.

**Figure 1:**
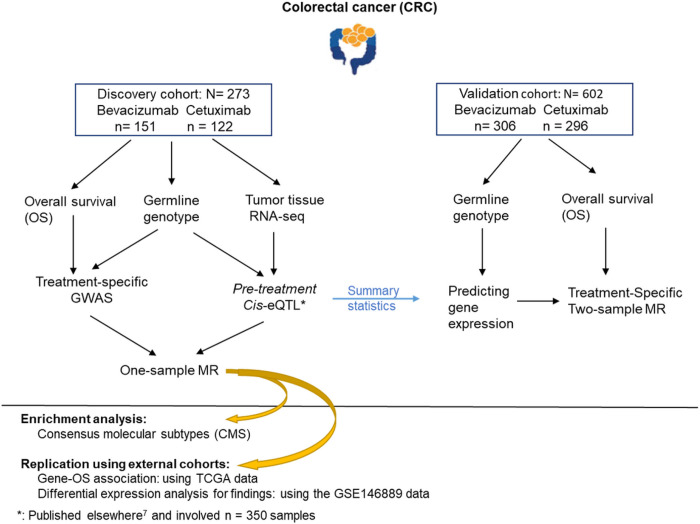
Study design and analytic workflow. The reported number of samples is after excluding patients with missing clinical outcomes or covariates.

The analyses reviewed above were adjusted for all RAS and BRAFv600E mutation statuses along with age and gender (Figure 1). Additionally, to enhance the pre*cis*ion of our findings, we accounted for the tumor microenvironment’s influence by adjusting for enriched immune cell abundances in the RNA-seq data.

### The effect of genes on treatment-specific OS

**Figure 2:**
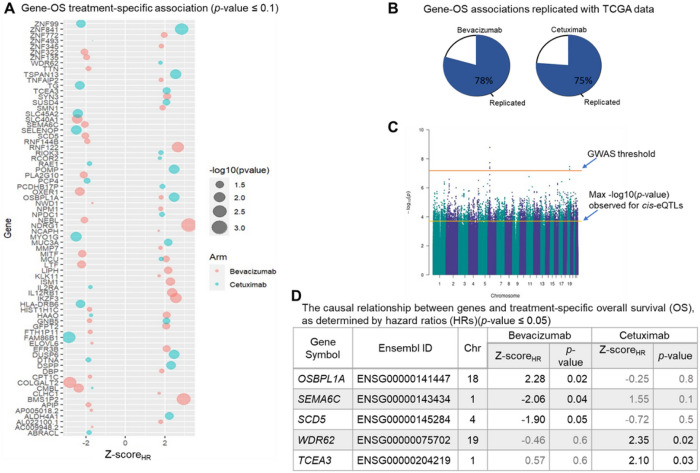
Gene-OS relationships. **A.** Genes associated with OS using the treatment-specific additive multivariable hazard model. **B** The pie charts represent the percentage of replicated gene-OS association using univariate analysis of the TCGA data. The rate of replication is relatively high even though the censoring rate of TCGA-COAD data is higher than the data in this study. **C** The Manhattan plot of the GWAS representing −*log10* (*p*-value) observed for genetic variants-OS associations. The GWAS threshold and the maximum −log(*p*-value) observed for the eQTL-OS associations are depicted on the plot. **D** Genes causally associated with treatment-specific OS based on a one-sample MR technique (*p*-value ≤ 0.05). The *p*-values under both treatments are presented for comparison.

To identify genes with causal effect on OS, we focused on 352 genes with eQTLs^7^, since they are good candidates for MR analysis. In the first step, we filtered these genes by investigating their association with OS. Out of the 352 genes with eQTLs, 79 genes were associated with OS (*p*-value < 0.1), including 47 under bevacizumab treatment and 32 under cetuximab (Figure 2A, **Table S1–2**)). We replicated the gene-OS associations using the TCGA data. The rate of replication was 78% and 75% for bevacizumab and cetuximab treatments, respectively (Figure 2B). Note that the replication rate is relatively high although the TCGA data is not a perfect replication set for the data in this study due to the higher censoring rate of the samples (**Figure S3**).

The association between genes and OS was conducted using multivariable time-variant additive hazard regression model which relaxes the assumption of the time-invariant effect of the genes on OS and allows for estimating the causal effects^8^ in the second step. Therefore, focusing on 79 genes associated with OS in the second step, we investigated the causal associations using one-sample MR technique with treatment-specific OS as outcomes, gene expressions as explanatory variables (exposures), and eQTLs as instrumental variables (IVs). To evaluate the validity of eQTLs as IVs, we assessed the lack of pleiotropic action by performing a genome-wide association study (GWAS) to find the association of the eQTLs with OS. The eQTLs did not show any significant association with OS at a level 1×10^−4^, which is much larger than the GWAS threshold (5×10^−8^) (Figure 2C, **S4**). Using the eQTLs as valid IVs, we predicted the expression levels of genes and estimated their causal effect on OS (Methods). We identified 5 genes with causal effect on OS (*p*-value < 0.05), including *OSBPL1A, SEMA6C*, and *SCD5* under bevacizumab treatment and *WDR62* and *TCEA3* under cetuximab (Figure 2D).

### Replication of the causal genes

**Figure 3:**
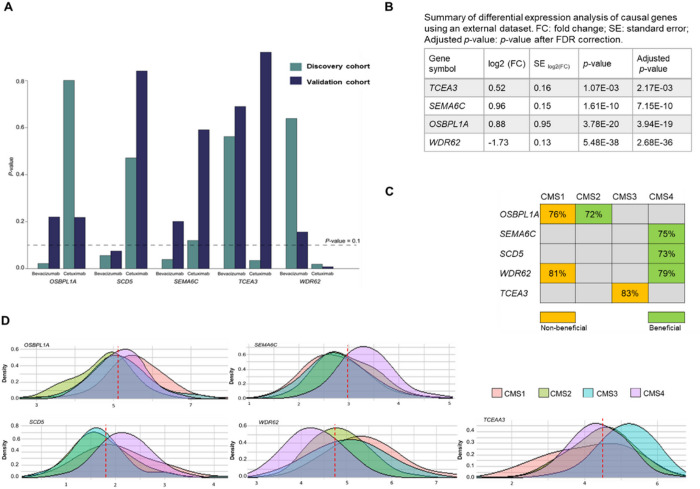
Replication of causal genes. **A.** Replication of causal effect of genes on treatment-specific OS in a validation cohort. The cyan bars represent *p*-values calculated in the discovery cohort using one-sample MR analysis for each treatment. The navy bars represent *p*-values calculated in the validation cohort using a two-sample MR study. The dashed line shows the *p*-value threshold level for significance. **B.** Summary statistics of differential expression analysis of causal genes in normal vs. CRC tumor tissues using an external cohort. **C** The enrichment of gene expressions causally associated with overall survival in each subtype is determined based on the high percentage of patients with beneficial or non-beneficial gene expression within that specific subtype (>70%). **D.** Illustrates the subtype-specific distribution of gene expression and their enrichment. The red dashed line represents the median expression level observed across all samples.

We reproduced the findings of one-sample MR analysis in the discovery cohort by performing a two-sample MR in the validation cohort. Using the eQTL summary statistics in CALGB-80405^7^ and the genotyping data in the validation cohort, we predicted expression levels in the validation cohort. We then estimated the effect of predicted genes on treatment-specific OS which represents the causal effect of the corresponding genes on OS (Methods). The *p*-values of significant genes-OS causal associations followed the same trend in the discovery and validation cohorts for bevacizumab vs. cetuximab, except *OSBPL1A*, (Figure 3A, **Table S3**). In addition, this analysis replicated that the causal effects of *WDR62* and *SCD5* were significant as identified in discovery cohort. The negative causal effect of *WDR62* on OS under cetuximab treatment (*p*-value: 0.008) compared to bevacizumab (*p*-value: 0.16) was replicated. Similarly, the positive causal effect of *SCD5* was also replicated, indicating its impact on OS under bevacizumab (*p*-value: 0.075) compared to cetuximab (*p*-value: 0.841).

In addition, we investigated whether genes causally associated with OS show differential expression between normal and tumor tissues. To assess this, we utilized GSE146889 data from the Gene Expression Omnibus database^9^ as an external cohort, which comprise 85 paired samples from normal and CRC tumor tissue. We applied DESeq2^10^ to 21,983 genes and corrected for multiple testing using Benjamini–Hochberg method (false discovery rate (FDR) < 0.05). We observed differential expression in *OSBPL1A*, *SEMA6C*, *WDR62*, and *TCEA3* (Figure 3B).

### Enrichment analysis for colorectal cancer subtypes

We investigated if the causal genes are enriched in CMSs of colorectal cancer: CMS1 (MSI Immune)-Tumors with high microsatellite instability (MSI-H) and strong immune activation; CMS2 (Canonical)-Tumors with features of traditional CRC, characterized by WNT and MYC signaling activation; CMS3 (Metabolic)- Tumors with metabolic dysregulation and KRAS mutations; CMS4 (Mesenchymal)- Tumors with prominent stromal infiltration, inflammation, and angiogenesis^3^. We dichotomized the expression levels of the casual genes into two categories: “beneficial” and “non-beneficial.” This dichotomization was based on the median expression level observed across all samples in our study. We then calculated the rate of patients with “beneficial” and “non-beneficial” expression levels within each subtype. We considered a threshold of 70%, and if over 70% of patients within a specific subtype showed either “beneficial” or “non-beneficial” gene expression, the subtype was considered enriched for the expression of the gene. Non-beneficial expressions of *OSBPL1A* and *WDR62* were enriched in CMS1 (rate 76% and 81%, respectively), whereas its beneficial expression levels were enriched in CMS2 (rate 72%). The CMS3 subtype exhibits an enrichment of non-beneficial levels of *TCEA3* (Rate: 83%), and the CMS4 subtype exhibits an enrichment of beneficial expression levels of *SEMA6C*, *SCD5*, and *WDR62* (Rate: 75%, 73%, 79%, respectively), (Figure 3C). The distribution of gene expressions specific to each subtype indicated an enrichment of gene expressions causally associated with overall survival in a specific subtype (Figure 3D).

## Discussion

Here, we integrated germline genotype and RNA-seq data from a randomized phase III trial and assessed the causal effect of gene expressions on OS of patients treated with either cetuximab or bevacizumab monoclonal antibodies. We identified 47 genes associated with OS of patients treated with bevacizumab and 32 genes in cetuximab, where three genes *OSBPL1A, SCD5*, and *SEMA6C* showed causal effect on OS in bevacizumab arm and two genes *TCEA3* and *WDR62* in cetuximab respectively. Replicating these results using external cohort represented differential expression of *OSBPL1A, SEMA6C, TCEA3*, and *WDR62*. We also replicated the causal effect of *WDR62* and *SCD5* on OS using a validation cohort with 602 additional samples.

*TCEA3* (Transcription Elongation Factor A3) is a protein-coding gene that is involved in the regulation of transcription, particularly in the elongation phase of transcription. Our study showed that the overexpression of *TCEA3* reduces the OS of patients treated with cetuximab. Additionally, more than 80% of patients with the CMS3 subtype exhibited high expression of *TCEA3*. In consistence with this finding here, the inhibition of *TCEA3* is suggested as an effective agent to enhance various chemotherapeutics-induced pyroptosis^11^.

*OSBPL1A* (Oxysterol-Binding Protein-Like 1A) is a gene that encodes a protein involved in the transport and regulation of lipids, specifically oxysterols. Oxysterols are oxidized derivatives of cholesterol and play roles in various cellular processes, including cholesterol homeostasis and lipid signaling which can affect the development and progression of various cancers, such as CRC. We observed negative impact of high expression of this gene on the OS of patients treated with bevacizumab, which was enriched among patients with CMS1 subtype.

Our study revealed that the upregulation of *SCD5* (Stearoyl-CoA Desaturase 5) potentially elongates OS in patients treated with bevacizumab, which is a VEGF inhibitor. *SCD5* is required for lipid synthesis, a key regulator of energy metabolism. Dysregulation of *SCD5* may play a role in dyslipidemia, characterized by abnormal lipid levels in the blood. Furthermore, dyslipidemia has been associated with CRC and tumor progression^12^. Moreover, the risk of dyslipidemia with the use of VEGF/VEGFR inhibitors has been observed^13^. Therefore, understanding the interplay between *SCD5*, dyslipidemia, and VEGF inhibition could yield valuable insights into CRC pathogenesis and therapeutic strategies.

Furthermore, our study showed that the overexpression of *WDR62* (WD repeat domain 62) shortened the OS of patients treated with cetuximab and may contribute to cetuximab-resistant CRC. *WDR62* is a scaffold protein involved in several important cellular processes, such as forming of the structure of the cell nucleus, regulation of gene expression, and ensuring proper division of cells during cell division. *WDR62* coordinates TNFα receptor signaling pathway to the JNK activation^14^. In addition, there is a complex interaction between TNF and EGFR, which is the targeted pathway of cetuximab^15^. *WDR62* also contributes to multidrug resistance of gastric cancer through activation of MAPK signaling^16^. In our study, overexpression of *WDR62* is enriched in CMS1 whereas its under expression is enriched in CMS4. Therefore, *WDR62* may be instrumental in understanding interactions between JNK signaling and specific CRC subtypes, potentially contributing to the development of more personalized and effective treatment strategies.

Collectively, our study underscores the utility of incorporating genotype data into gene-OS investigations and in increasing the likelihood of discovering novel biomarkers while shedding light on unrecognized mechanisms specific to cancer subtypes.

## Materials And Methods

### Data

Patients in this study were drawn from the Cancer and Leukemia Group B (CALGB; now a part of the Alliance for Clinical Trials in Oncology) and SWOG 80405 (Alliance) trial. The trial was initiated in September 2005 with a total of 2,326 patients randomized to the three treatment arms (bevacizumab, cetuximab, or their combination in addition to chemotherapy with FOLFIRI or FOLFOX).

#### Genotyping:

DNA was extracted from peripheral blood. The first genotyping batch was performed on the Illumina HumanOmniExpress-12v1 platform at the Riken Institute (Tokyo, Japan) and included 731,412 genotyped variants. The second genotyping batch was performed on the Illumina HumanOmniExpress-8v1 and included 964,193 SNPs. A total of 719,461 SNPs from HapMap from batch 1 were also on the chip from batch 2. The QC was performed to remove SNPs with mismatched annotation between the two platforms, genotyping call rates less than 99%, departure from Hardy–Weinberg equilibrium (*P*<10^−8^), allele frequencies less than 0.05, and individuals with genotyping call rate < 0.90. Passing the filters, 540,021 SNPs genotyped for 1,165 samples were remained^17^.

#### Tumor RNA sequencing:

Tumor RNA was extracted from formalin-fixed paraffin-embedded (FFPE) tumor blocks (96% primary, 2% metastatic, 2% unknown) from 584 CALGB/SWOG 80405 patients at the baseline. TruSeq RNA Access target enrichment and library preparation protocol was performed using 250 ng of template RNA. Sequencing was done using synthesis chemistry targeting 50 M reads with a read length of 2×100 bp per sample on the HiSeq 2500. Data processing was conducted using standard procedures^18^.

#### Clinical outcomes and covariates:

The primary endpoint of OS was calculated from the time of study entry to death or last known follow-up for those without reported death. The median follow-up of 640 samples in bevacizumab, cetuximab was 65.7 months (95% confidence interval, 63.5–70). In addition, *BRAF* and all *RAS* mutation status were determined by BEAMing (beads, emulsion, amplification, magnetics; Hamburg, Germany) technology^19^ and included in the analysis as covariates in addition to age and gender.

## Methods

### Data preprocessing

Among 584 samples with RNA-seq data, 86% were Caucasian, 9% African American, 5% from other ethnicities. Therefore, we focused on primary tumor samples from Caucasians to avoid analysis being confounded due to population stratification. We excluded genes with low expression variation across samples (standard deviation < 0.5) and genes with low counts across the samples (> 30% zeros). The remaining was 8301 genes for the analysis and we applied upper quartile normalization to make gene expression values comparable among different samples. We removed duplicated samples (n=5) and tumors with low gene expression across the genome (> 50% genes with zero counts, n=1). We then transformed the RNA-seq data into the log2 scale for the analysis. We performed principal component analysis to assess for batch effect or hidden population stratification in RNA-seq data (**Figure S5**). To verify the self-reported gender, we applied k-mean clustering using the expression of genes in chromosome Y, resulting in 5 samples with mismatched biological gender and recorded gender^7^ (**Figure S6**).

We used 1,055 tumor samples (Caucasian), genotyped at 540,021 SNPs for imputation. We used phased haplotypes from the Haplotype Reference Consortium (HRC) panel through the Michigan server^20^. Phasing was done using Eagle v2.4 algorithm^21^. The HRC panel combines sequence data across > 32,000 individuals from >20 medical sequencing studies. The imputed genotype data with imputation score > 0.7 and minor allele frequency (MAF) > 0.05 included 5,539,144 common SNPs. These SNPs were used in all the analyses.

### Immune cell type abundance

Since the RNA-seq data in this study has been generated from heterogeneous tumor samples composed of multiple cell types, correcting for the abundance of different cell types in the data is crucial to avoid the analysis being confounded. We estimated the abundance of immune cell types in our RNA-seq data using CIBERSORTx^22^with the validated leukocyte gene signature matrix as a reference. We defined a cell phenotype to be enriched in our data if at most 30% of its estimated scores across samples are zero and its standard deviation is greater than 0.12. As a result, 9 hematopoietic cell phenotypes were enriched in our data: naive and memory B cells, plasma cells, CD8+ T cells, resting and activated memory CD4+ T cells, M0 and M2 macrophages, and activated mast cells2^7^.

### *cis*-eQTL analysis

To identify germline genetic variants associated with tumor gene expression, we previously focused on *cis*-eQTL^23,24^. For all pairs of genes and SNPs within 1 Mb upstream and downstream of the gene’ transcription start site (TSS), we applied a linear regression model while accounting for covariates (gender, age, BRAF V600E mutations and all RAS mutation status, batch effects, enriched cell type abundance). We performed *cis*-eQTL mapping using FastQT^25^. We applied the adaptive permutation mode of FastQTL while setting for 10,000 permutations and selected genes with at least one *cis*-eQTL with an adjusted *p*-value < 0.05 at the gene level. The result of *cis*-eQTL analysis is published elsewhere^7^ (**Figure S7–8**). Hereafter, for simplicity, we use eQTL to refer to *cis*-eQTL.

### Mendelian randomization (MR) study.

To apply MR technique and fulfill the assumptions, we take the following steps focusing on the genes with eQTLs.

#### 1. Gene-OS association:

To investigate if genes with eQTL are associated with OS (i.e, the outcome), we applied multivariable time-variant additive hazard regression model. This additive hazard regression model relaxes the assumption of the time invariant effect of the genes on OS and allows for estimating causal effects^8^. To enhance the feasibility of conducting multivariable analysis and to prevent overfitting, we employed *k*-mean clustering and clustered all genes in 4 clusters^7^. Using these clusters, we partitioned 352 genes into 4 clusters and performed multivariable analysis by including all the genes in each cluster in the model. The analyses were performed separately for the patients under bevacizumab treatment and cetuximab treatment while accounting for the tumor microenvironment’s influence by adjusting for enriched immune cell abundances in our RNA-seq data in addition to the set of covariates gender, age, BRAF V600E mutations and all RAS mutation status. Gene associated with OS (*p*-value < 0.1) were considered for causal study using the MR technique reviewed in the next steps.

#### 2. Instrumental variables and the lack of pleiotropy assessment:

MR techniques aim at distinguishing causations from associations in observational studies by using genetic factors as instrumental variables (IVs). All 352 genes considered in the step 1 for association study were genes with eQTLs. We considered eQTLs as potential IVs and staringly assessed the lack of pleiotropic action. We conducted a GWAS and estimated the effect of IVs on OS via a Cox proportional hazard model while adjusting for covariates gender, age, tumor location, all-RAS, and KRAS mutation as well as the first principal component accounting for the batch effect (**Figure S10**). The significance threshold in GWAS is typically set at a *p*-value of less than 5×10^−8^. Any association above this threshold for IV-OS associations can be assumed as a lack of pleiotropy. However, we employed a stringent threshold of *p*-value < 1×10^−4^ and excluded IVs with *p*-values < 1×10^−4^ to ensure that the assumption of lack of pleiotropy holds.

#### 3. Gene-OS causal association:

We evaluated the causal impact of associated genes with OS by predicting the expression level of each gene using its IVs. If the predicted expression value is associated with OS, the corresponding gene is considered as a putative cause of OS.

To predict the expression level of a gene, we initially clustered the IVs of the gene based on their correlation (r^2^ > 0.1 within each cluster) using hierarchical clustering. We then selected one IV from each cluster as the proxy to avoid overfitting. We then predicted the expression level of each gene $\left(\overset{ˆ}{g}\right)$ as follows

$${\overset{ˆ}{g}}_{i}=Q_{i}W_{i}.$$

Here $Q_{i}$ is an $n\times p_{i}$ matrix of IVs where n is the sample size and $p_{i}$ is number of IVs for *i*th gene $\left(g_{i}\right)$, and $W_{i}$ is a $p_{i}\times 1$ vector of the estimated cofficients from the eQTL analysis. We investigated the the gene-OS relationship using ${\overset{ˆ}{g}}_{i}\text{s}$ in a multivariable additive hazard model described earlier. The model included all predicted genes. The genes with the significant $\overset{ˆ}{g}-\text{OS}$ relationship (*p-*values < 0.1) were considered as genes with causal effect on OS.

### Replication of one-sample MR findings by a two-sample MR study:

For the replication of causal genes from the previous section, we used 602 samples in the validation cohort (296 under bevacizumab treatment and 306 under cetuximab treatment) with genotype and clinical outcome and no RNA-seq data. We first compared the clinical characteristics and demography of patients in discovery and validation cohorts^1^ (**Table S4**) to ensure compatibility of the cohorts. We then predicted gene expression levels of the causal genes in the validation cohort using summary statistics of eQTLs from discovery cohort as:

$${\overset{ˆ}{g}}_{i}^{*}=Q_{i}^{*}{\left(Q_{i}^{T}Q_{i}\right)}^{-1}Q_{i}^{T}g_{i},$$

where $Q_{i}$ is an $n\times p_{i}$ matrix of eQTL where n is the sample size discovery cohort (with eQTL) and $p_{i}$ is the number of eQTLs selected as IVs for the *i*th gene $\left(g_{i}\right)$, and $Q_{i}^{*}$ is a $n^{*}\times p_{i}$ matrix where $n^{*}$ is the sample size in validation cohort. If the predicted expression levels $\left({\overset{ˆ}{g}}_{i}^{*}\right)$ significantly affected OS via treatment-specific additive hazard model, we considered it a replication for the one-sample MR. This analysis has some similarities with transcriptome-wide association studies (TWAS). But the main difference is that, in the two-sample MR analysis, genetic variants as predictors satisfy MR assumptions, which might not be the case in TWAS.

## Data Availability

The gene expression data generated in this study are publicly available in Gene Expression Omnibus at GSE196576.
